# Interaction between the gut microbiome and mucosal immune system

**DOI:** 10.1186/s40779-017-0122-9

**Published:** 2017-04-27

**Authors:** Na Shi, Na Li, Xinwang Duan, Haitao Niu

**Affiliations:** 1 Institute of Laboratory Animal Sciences, Chinese Academy of Medical Sciences (CAMS) and Comparative Medicine Center, Peking Union Medical Collage (PUMC), Key Laboratory of Human Disease Comparative Medicine, Ministry of Health, Beijing, 100021 China; 2 Department of Rheumatology, the Second Affiliated Hospital of Nanchang University, Nanchang, 330006 China

**Keywords:** Microbiome, Immunity, Inflammation

## Abstract

The gut microbiota, the largest symbiotic ecosystem with the host, has been shown to play important roles in maintaining intestinal homeostasis. Dysbiosis of the gut microbiome is caused by the imbalance between the commensal and pathogenic microbiomes. The commensal microbiome regulates the maturation of the mucosal immune system, while the pathogenic microbiome causes immunity dysfunction, resulting in disease development. The gut mucosal immune system, which consists of lymph nodes, lamina propria and epithelial cells, constitutes a protective barrier for the integrity of the intestinal tract. The composition of the gut microbiota is under the surveillance of the normal mucosal immune system. Inflammation, which is caused by abnormal immune responses, influences the balance of the gut microbiome, resulting in intestinal diseases. In this review, we briefly outlined the interaction between the gut microbiota and the immune system and provided a reference for future studies.

## Background

The mammalian gut contains a microbial community, defined as the microbiome, which includes bacteria, viruses, fungi, etc. Microbial genome sequences contain 3 × 10^6^ genes, which is approximately 150-fold the length of the human genome [1]. In recent decades, next generation sequencing technology has contributed to understanding the intricate relationship between the microbiome and related diseases. 16S rRNA sequencing results showed that *Firmicutes* and *Bacteroidetes* make up approximately 92% of the human microbiome [2]. Gut microbiota include 1,000 to 1,500 bacterial species; however, an individual contains only approximately 160 bacterial species, indicating that the composition of the microbiome is substantially different between individuals and is related to environmental changes and genetic inheritance [3, 4]. Environmental factors play a very important role in the gut microbiome. Even mice with the same genotype housed in separate cages within the same facility show different microbiota compositions [5]. The composition of the mouse gut microbiome is mainly influenced by variations in diet, age and inflammation [5, 6]. A review of studies also showed that the composition of the gut microbiome in an eczema population is influenced by environmental factors, including pregnancy duration, delivery method, feeding type, rearing style, number of siblings, lifestyle, etc. [7]. The intestinal microbiome, a microbial organ that is shaped in combination with the host’s genotype, responds to the growth process and environmental exposure. The coordinated interactions between intestinal microbial populations contribute to maintaining intestinal homeostasis and play an important role in the immune process.

The symbiotic relationship between microbiota and the host is mutually beneficial. The host provides an important habitat and nutrients for the microbiome, and the gut microbiota support the development of the metabolic system and the maturation of the intestinal immune system by providing beneficial nutrients, e.g., by the synthesis of vitamins [8] and short chain fatty acids (SCFAs) [9, 10]. Therefore, the interaction between the microbiome and intestinal immune system is critical to maintain mucosal homeostasis. However, when the balanced gut microbial communities change, dysbiosis causes intestinal diseases [11]. Microbial colonization depends on the development of the immune system. The rapid colonization of microbiota in the neonatal gastrointestinal tract plays a vital role in the development of the gut immune system [12]. However, studies in Germ-Free (GF) animals revealed that the lack of gut microbiota caused a significant immune system deficiency. Furthermore, the dysbiosis of gut microbiota has been closely linked to several diseases, such as obesity [13], type 2 diabetes [14], hypertension [15], necrotizing enterocolitis (NEC) [16], and inflammatory bowel diseases(IBD) [17], etc.

The aim of this review is to briefly summarize the interaction between the gut microbiome and mucosal immune system, as well as the development of autoimmune diseases.

## The intestinal mucosal immune system

The immune system is regulated by immune organs, immune cells, soluble cytokines and cell receptors. The intestine mucosal immune system consists of three different mucosal lymphoid structures: Peyer’s patches, the lamina propria and the epithelia [6].

The mucus layer on the surface of epithelial cells is the first line of defense in the organism’s physiological barrier. In the small intestine epithelia, Paneth cells located at the base of crypts are capable of secreting antimicrobial peptides (AMPs) in response to bacteria or pathogens in the gut lumen and contribute to intestinal innate host defense [18]. The AMPs include α-defensins (HD5 and HD6 in humans and cryptdins in mice), RegIII and lysozymes, etc. [19]. The mucus layer and AMPs constitute the mucosal barrier to prevent the invasion of symbiotic bacteria. Pioneering studies have discovered an important role for AMPs in the host mucosal defense, indicating that they directly affect the microbiome in the intestinal lumen [20–22]. AMPs can exert antimicrobial activities to kill microorganisms *in vitro* [18]. RegIII specifically targets Gram-positive bacteria. Additionally, bacteria and bacterial antigens increased the expression of RegIIIγ [23], cryptdin [24], and human β-defensin 2 [25]. Moreover, RegIIIβ was significantly increased, and it was released into the gut lumen in response to infection [26]. RegIIIγ played a vital role in segregating the bacteria from the intestinal epithelium, and the absence of RegIIIγ led to increased bacteria colonization on the epithelium and the activation of adaptive immunity [27].

Epithelial cells are the second physical barrier of the intestinal mucosal immune system, and they directly participate in the immune surveillance of the gut. Epithelial cells are not only involved in the direct defense of microorganisms; they also send signals to the mucosal immune system by producing cytokines and chemokines [11]. In response to stimuli, a class of innate lymphoid cells (ILCs) located in the epithelial cells can be activated to produce cytokines, which play a defensive or a pathogenic role in inflammation; this response is closely related to the control of intestinal homeostasis in mammals. IL-22 is produced by ILCs and promotes homeostasis and healing during infection in the gut [28, 29]. IL-22 is also capable of inducing epithelial cells to produce RegIIIα, which bonds bacterial peptidoglycan carbohydrates and kills targeted Gram-positive bacteria [30]. The microbiome produces metabolites, such as butyrate and tryptophan decomposition metabolites that are able to enhance gut integrity and stimulate innate lymphoid cells group 3 (ILC3) to produce IL-22 [9]. Intraepithelial lymphocytes (IELs), which consist of αβ^+^ and γδ^+^T cell populations, play an important role in defense and pathogenesis during inflammation. When IELs are activated, they express cytokines, such as IFN-γ and keratinocyte growth factor, to protect epithelial cells from injury [31–33]. The level of IFN-γ, which is produced by IELs, is closely linked to IBD [34]. Dendritic cells (DCs) are responsible for the immune system’s ability to effectively recognize and eliminate exogenous pathogens. DCs have the ability to continuously pass antigens through the barrier to mucosal-associated lymphoid tissue or to drain lymph nodes [35]. Additionally, DCs open the tight junction between the intestinal epithelium and directly enter the lumen to phagocytose *Salmonella* and *E. coli* [35]. At the steady state, DCs regulate intestinal immune tolerance by promoting the differentiation of CD4^+^ T cells toward regulatory T cells (Tregs) [36] and activating the Tregs through the non-classical autophagy pathway [37]. Sierro et al. [38] further confirmed that the *Salmonella* flagella protein specifically induced the up-regulated expression of CCL20 and led to DC migration. Pro-inflammatory T helper (Th) cells play an important role in autoimmunity by eliminating pathogens during the host defense reaction and inducing tissue inflammation, which leads to subsequent tissue destruction. Tregs is a major regulatory component in immune tolerance and inflammation. Therefore, the dysregulation of Tregs and pro-inflammatory Th cells in the gut are closely associated with intestinal autoimmunity, such as in IBD [36]. In addition, secretory cells in the epithelial cell layer can synthesize and secrete proteoglycans to form mucus and other cells involved in auxiliary microbial defense [11]. Epithelial cells also express various pattern recognition receptors (PRRs), including Toll-like receptors (TLRs) and nucleotide-binding oligomerization domain-containing protein 2 (NOD2), which also produce chemokines for bone marrow cells and lymphocytes upon anti-inflammatory stimuli [39–41]. TLRs are a group of important PRRs play a vital role in the innate immune system [40]. Microbes can recognize immune regulators, such as chemokines, pro-inflammatory cytokines and anti-inflammatory cytokines, through PRR, and these regulators play important roles in autoimmunity and adaptive immunity.

The lamina propria, which consists of B and T cells, resides in the lower layer of intestinal epithelial cells. T cells quickly respond to the signal from the lumen environment and initiate inflammatory and anti-inflammatory responses. The intestinal microbiome promoted the differentiation of naive CD8^+^ T cells toward CD4^+^ T cells [42]. The lamina propria CD4^+^ T cells secreted IL-17 and IL-22, which were involved in regulating intestinal inflammation [43]. Intestinal epithelial cells produce IL-17, which can induce the expression of chemokines, such as CXC and CC chemokines [44]. Peyer’s patches, the location for IgA-producing B cell maturation, are distributed along the small intestine with counts of 100–200 in humans and 6–12 in mice [45]. These patches contribute to generating B cells and plasma cells. Activated B cells in Peyer’s patches consistently generate IgA-producing plasma cells for T cell-dependent and T cell-independent responses in the gut [46, 47]. Secreting IgA is their major contribution to protecting the gut barrier [48]. After activation, T cells and B cells return to the lamina propria, functioning as part of the specific immune response [11]. Intestinal microfold (M) cells are epithelial cells that are primarily present in Peyer’s patches of the small intestine and shuttle antigens into the Peyer’s patch for appropriate immune responses. Most recently, Sialic acid-binding immunoglobulin-like lectin F (Siglec-F) expression was identified on mouse M cells in the small intestine; Siglec-F functions in antigen transportation in the gut [49].

## Gut microbiome and mucosal immunity

Over the course of evolution, the microbiome maintains symbiosis with the gut environment. The human gut provides nutrients and a breeding environment for intestinal microflora; in turn, intestinal microflora assists in carbohydrate fermentation and synthesize vitamins by reducing intestinal permeability and increasing the epithelial defense mechanism to form a mucosal barrier [50]. The intestinal mucosal immune system constitutes the largest immune component in vertebrates, functioning closely with the intestinal microbiome. The balance of the intestinal mucosa immune system plays a key role in host homeostasis and defense. Studies on GF mice suggested that the intestinal microbiome plays a vital role in the formation of mucosal immunity. Compared with specific pathogen free (SPF) animals, GF animals produce fewer IELs [51] and have significantly reduced IgA-secreting plasma cells in the lamina propria [52], as well as fewer Tregs [53]. Angiogenin-4 (Ang4) is a class of microbicidal proteins in Paneth cells and can be secreted into the gut lumen against microbes. Real-time quantitative RT-PCR suggested that the mRNA expression level of Ang4 markedly decreased in GF mice compared with conventional mice [54]. This result indicates that gut microbiota is required for mucosal immunity. Additionally, Peyer’s patches in GF mice contain a smaller germinal center than in conventional mice [9]. The intestinal mucosa is the main site for microbiome-host interactions. A recent study showed that IgA in the feces significantly increased after treatment with prebiotics, while the expression of pro-inflammatory factor in the mesenteric lymph nodes and Peyer’s patches was significantly reduced. Additionally, the IL-10, CXCL-1 and Mucin-6 genes were up-regulated, while the colonic mucosa 4, IFN-$\gamma$, GM-CSF and IL-1$\beta$ genes were down-regulated in the ileum [48]. These results indicated that the gut microbiome affect intestinal mucosal immune balance.

The dynamic interaction between the microbiome and environmental factors shapes mucosal and systemic immunity. Diet and exogenous substrates are the key regulatory factors of the intestinal microbiome. In obese subjects, the ratio of *Bacteroidetes/Firmicutes* was reported to be decreased [55]. High fat and high sugar diet feeding changed the composition and diversity of the gut microbiome in mice, leading to altered SCFA production [56]. In healthy individuals, the microbiome primarily consists of 4 groups of bacteria: *Firmicutes*, *Bacteroidetes*, *Proteobacteria* and *Actinobacteria* [57].

Carasi et al. [48] found that LPS induced a significant decline in the production of IL-6 and GM-CSF in *Lactobacillus kefiri*-treated mice, indicating that *Lactobacillus kefiri* is an important factor of intestinal inflammatory dysbiosis. *Bacteroides fragilis*, a type of symbiotic bacteria, can produce polysaccharide A with anti-inflammatory effects by inhibiting IL-17 production and enhancing the activity of intestinal Tregs [58, 59]. Polysaccharide A regulates CD4^+^ T cell transformation toward Foxp3^+^ Tregs in a TLR2-dependent manner [59]. Subsequently, Tregs produce anti-inflammatory IL-10 in response to defend inflammatory injuries [59, 60]. The colonization of *Clostridium* clusters IV and XIVa in the large intestine enhance the level of TGF-β_1_ and promote IL-10-expressed Foxp3^+^ Tregs [61, 62]. However, *Clostridium* clusters IV and XIVa declined in IBD. An analysis of dsDNA virus-like particles in IBD patients suggested that the *Caudovirales* bacteriophages remarkably expanded, and this expansion was associated with a significant alteration of bacteria [63]. Hence, the complex interaction between the microbiome and its host maintains a spatial separation of the microbiome and intestinal epithelial cells, which promotes host homeostasis. The effect of segmented filamentous bacteria (SFB) on promoting the Th17 response has drawn substantial attention. SFB are closely related to intestinal inflammation [64, 65]. The colonization of SFB promoted systemic Th17 cell activation and triggered arthritis in GF K/BxN mice [66], as well as accelerated experimental autoimmune encephalomyelitis [67].

## The dysbiosis of the gut microbiome induces intestinal diseases

The intestinal microbiota and mucosal immunity constantly interact to achieve intestinal homeostasis. However, once the balance is broken, dysfunction of the intestinal immune system will trigger a variety of diseases, such as IBD. IBD is a heterogeneous disease influenced by genetic, environmental and microbial factors, leading to intestinal inflammation by triggering an abnormal immune response. Crohn’s disease (CD) and ulcerative colitis (UC) are typical IBDs [50]. Studies have shown that genetic susceptibility was not sufficient to trigger IBD, the concordance of which was only 35% for CD and 16% for UC in monozygotic twins [68]. This result indicated that in addition to genetic factors, environmental and microbial factors are also important for IBD pathogenesis. Studies have shown that intestinal dysbiosis led to an abnormal adaptive immune response that increased IBD inflammation and the destruction of the gastrointestinal tract [69, 70]. The rapid development of next generation sequencing technology has provided further information on the human genome and the composition of the intestinal microflora genome, and it has aided in the determination of which species of intestinal microorganisms are related to disease development [2, 37]. Therefore, intestinal microflora manipulation has been a powerful preventive and therapeutic intervention against inflammation. Among these, fecal microbiota transplantation is an effective treatment for IBD [71]. Recent studies have shown that one of the important genes for regulating the pathogenesis of IBD, Caspase recruitment domain family member 9 (*CARD9*), was responsible for mediating intracellular signals to trigger inflammation. The intestinal microbial structure was altered and susceptible to intestinal fungus infection in *CARD9* knockout mice, indicating that the deletion of *CARD9* led to IBD dysbiosis [72]. In patients with mild to moderate colitis, the production of IL-22 by ILC3 was elevated when exposed to excreta, suggesting the involvement of gut microbiota [73]. Additional studies have also found a relationship between the abnormal expression of miRNA and the development of IBD. MiR-19b expression was found to be significantly reduced in CD [74]. Bioinformatics analysis showed that a suppressor of cytokine signaling (SOCS3) was considered the possible target of miR-19b. By inhibiting SOCS3, MiR-19b regulates intestinal epithelial cells to produce cytokines, thereby inhibiting the inflammatory response and eventually preventing the onset of CD [74].

Altered microbiota diversity was found in IBD, and it was reflected by a decline in commensal bacteria, such as *Firmicutes* and *Bacteroidetes*, and an increase in detrimental bacteria, such as *Proteobacteria* and *Actinobacteria* [17]. Due to the decreased microbial diversity in IBD, the ability of microbiota to adapt to environmental changes and to defend against natural disturbances is impaired. Active bacterial products can regulate the inflammatory response in IBD. For example, IL-10 deficiency was found to be associated with early-onset IBD [75, 76]. SCFAs are dietary fiber produced by gut bacteria fermentation. Studies of fecal samples from IBD patients showed that SCFA levels were remarkably changed, supporting the important role of SCFAs in IBD [77]. SCFAs regulate certain inflammatory responses by binding GPR43 [78]. Additionally, SCFAs regulate colonic Treg cell homeostasis by restoring the colonic size and function of the Treg cell pool in GF mice [79].

Gut microbiota produce many immunogenicity substances. For example, complex lipopolysaccharides on the cell surface of Gram-negative bacteria caused a fecal immune response. In some cases, immunogenic substances pass through the intestinal wall, especially when the barrier is destroyed, causing further damage [80]. *Bacteroides fragilis* found in the human intestine plays a positive regulatory role in the human immune system. In GF mice, *Bacteroides fragilis* promoted the Foxp3^+^ T cells to produce anti-inflammatory cytokines in the gut [59]. Recent studies showed that intestinal microflora enveloped with IgA from IBD patients promoted the sensibility of dextran sodium sulfate-induced colitis in GF mice. Intestinal microflora, especially *enterobacterium*, promoted host IgA effects [81]. These studies highlight the complex interactions between intestinal microflora and IgA in the pathogenesis of intestinal inflammation.

## Effects of gut microbiome and mucosal immunity in autoimmune diseases

The pathogenetic mechanism of systemic autoimmune diseases remains unclear; genetic and environmental factors may have certain effects. A gut microbiome challenge may initiate autoimmune diseases. Studies have shown that some autoantibodies, such as antinuclear antibodies, anti-double-stranded DNA in systemic lupus erythematosus (SLE), and rheumatoid factor, anti-citrullinated protein antibodies (ACPAs) in rheumatoid arthritis (RA) could be detected before the onset, indicating that the microbiome plays an important role in the development of autoimmune diseases, especially *Porphyromonas gingivalis* (*P.gingivalis*) [82–84]. Hevia et al. [85] recently found that the ratio of gut *Firmicutes*/*Bacteroidetes* decreased in SLE patients, indicating that the dysfunction of mucosal immunity in SLE patients may influence the gut microbiome community. Similarly, *P. gingivalis* was shown to be the potential initiator, causing RA to form ACPAs [84]. Recent studies have shown a close relationship between microbiota and RA, including *Mycoplasma* [86]*, Proteus* [87], *Escherichia* [88], *Haemophilusspp, Lactobacillussalivarius*, etc. [89]. 16S rRNA analysis showed that *Lactobacillus* significantly increased in the fecal microbiota of RA patients compared to the control [90]. Compared to RA patients with long-term treatment, an increase in *Prevotella* and a decline in *Bacteroides* were found in early RA patients [91], indicating the influence of *Prevotella*in RA disease development. The functional analysis of *Prevotella*-dominating metagenomics showed that the purine metabolic pathways significantly declined, which may have affected the therapeutic efficiency of methotrexate in RA [91]. Studies also showed that the microbial components in the terminal ileum significantly changed in ankylosing spondylitis ﻿(AS); the abundance of five bacterial species significantly increased, including *Lachnospiraceae*, *Ruminococcaceae*, *Rikenellaceae*, *Porphyromonadaceae* and *Bacteroidaceae*. Conversely, *Veillonellaceae* and *Prevotellaceae* declined [92]. Hence, the dysbiosis of gut microbiota is closely associated with autoimmune diseases.

## Conclusions

In summary, intestinal microbiota coordinates to shape host immunity and contribute to maintaining intestinal homeostasis and inhibiting inflammation. Recent data have shown the pivotal role of intestinal microbiota in mucosal immunity. An impaired interaction between intestinal microbiota and mucosal immune system is associated with the pathogenesis of inflammatory diseases, such as IBD, RA, SLE, AS, etc., and it highlights the importance of exploring the function of microbiota in such diseases. Thus, intestinal microbiota has become effective targets for the development of new diagnostic methods. Balancing the gut microbiome will likely represent an effective treatment for chronic inflammatory diseases.

## Abbreviations

ACPAs: Anti-citrullinated protein antibodies
AMP: Antimicrobial peptides
Ang4: Angiogenin-4
AS: Ankylosing spondylitis
CARD9: Caspase recruitment domain family member 9
CD: Crohn’s disease
DC: Dendritic cell
GF: Germ-free
IBD: Inflammatory bowel disease
IEL: Intraepithelial lymphocyte
ILC: Innate lymphoid cell
NEC: Necrotizing enterocolitis
NOD2: Nucleotide-binding oligomerization domain-containing protein 2
*P.gingivalis*: *Porphyromonas gingivalis*
PRR: Pattern recognition receptor
RA: Rheumatoid arthritis
SCFA: Short chain fatty acid
SFB: Segmented filamentous bacterium
Siglec-F: Sialic acid-binding immunoglobulin-like lectin F
SLE: Systemic lupus erythematosus
SOCS3: Suppressor of cytokine signaling
SPF: Specific pathogen free
Th: T helper
TLR: Toll-like receptors
Treg: Regulatory T cell
UC: Ulcerative colitis
